# Cocaine-Induced Four-Extremity Ischemia Caused by a Hypercoagulable State

**DOI:** 10.7759/cureus.44862

**Published:** 2023-09-07

**Authors:** Victoria Echevarria, Alexandra C Echevarria, Damian Casadesus

**Affiliations:** 1 Internal Medicine, American University of the Caribbean, Sint Maarten, MAF; 2 Internal Medicine, Nova Southeastern University Dr. Kiran C. Patel College of Osteopathic Medicine, Davie, USA; 3 Internal Medicine, Jackson Memorial Hospital, Miami, USA

**Keywords:** limb thrombosis, cocaine hypercoagulable state, limb ischemia, drug-induced ischemia, cocaine-induced thrombosis

## Abstract

The use of cocaine is associated with serious complications including coronary vasospasm and myocardial, renal, intestinal, and neurological ischemia. Among these feared complications lies limb ischemia which is a rare potential side effect of chronic cocaine use. We present the case of a 50-year-old female with an extensive history of cocaine use who developed ischemia in all four limbs. Imaging studies revealed pulmonary emboli, multisystem thromboses, and microhemorrhages in the brain. Laboratory studies were significant for leukocytosis, thrombocytopenia, schistocytes on blood smear, and normal rheumatologic and hematologic studies. The patient was diagnosed with cocaine-induced thrombotic microangiopathy and she was treated symptomatically and with continuous heparin infusion. However, she ultimately requested to be discharged home and was lost to follow-up. Cocaine-induced thrombotic microangiopathy has been reported in only a few other patients to date and although there are some theories describing the possible pathophysiology, a clearly defined explanation has not yet been widely accepted.

## Introduction

Cocaine has been reported as one of the most used illicit drugs and its use was mentioned in 30% of all drug-related hospital visits in 1999 [1]. Although unfortunate, medical examiners frequently identify cocaine in drug-related deaths, and in 2011, 40.3% of patients who tested positive for illicit drugs during emergency room visits also tested positive for cocaine. The use and abuse of illicit drugs has been steadily increasing in recent decades and according to the Center for Disease Control and Prevention, cocaine-induced deaths increased from 19,927 in the year 2020 to 24,538 in the year 2021.

Chronic cocaine abuse has been reported to have several deleterious effects on vascular structures mainly due to its propensity for vasoconstriction. These complications include but are not limited to ischemic vascular disease, organ damage, atherosclerosis, and hemodynamic instability [2]. One uncommon complication of chronic cocaine use is arterial thrombosis which occurs within small-diameter vessels, such as the cerebral and coronary arteries, leading to ischemia. This is thought to be a result of a cocaine-induced increase in sympathetic activity causing catecholamines to concentrate in the post-synaptic cleft due to inhibition of re-uptake. The catecholamines then promote platelet aggregation via stimulation of alpha-adrenoreceptors which creates thrombotic occlusion in the small-diameter vessels [2]. However, there are researchers who suggest alternate sources of thrombus formation including interactions of cocaine with arachidonic acid or thromboxane in contrast to adrenoreceptors [2].

Cocaine’s role in causing vascular and endothelial dysfunction can further affect various organ systems via vasospasms and the aforementioned vascular thrombosis. Often one of the first presenting symptoms in cocaine-induced ischemia is a diffuse rash known as retiform purpura. The rash is non-blanching and appears as a net-like pattern on examination. A biopsy of the skin reveals intravascular occlusion without signs of vasculitis. This vasculopathy can affect the ears, face, and/or extremities [3]. Secondary effects of these complications include splenic infarction, portal vein thrombosis, extremity ischemia, and myocardial infarction [4]. Long-term use of cocaine can result in multi-system involvement which can eventually end in death if not managed properly. Treatment options depend on the degree of organ involvement but generally include cessation of the offending agent, blood pressure management, and supportive care [5]. Herein, we present a woman who developed cocaine-associated severe limb ischemia with multiple thromboemboli and gangrene of all four limbs.

## Case presentation

**Figure 1:**
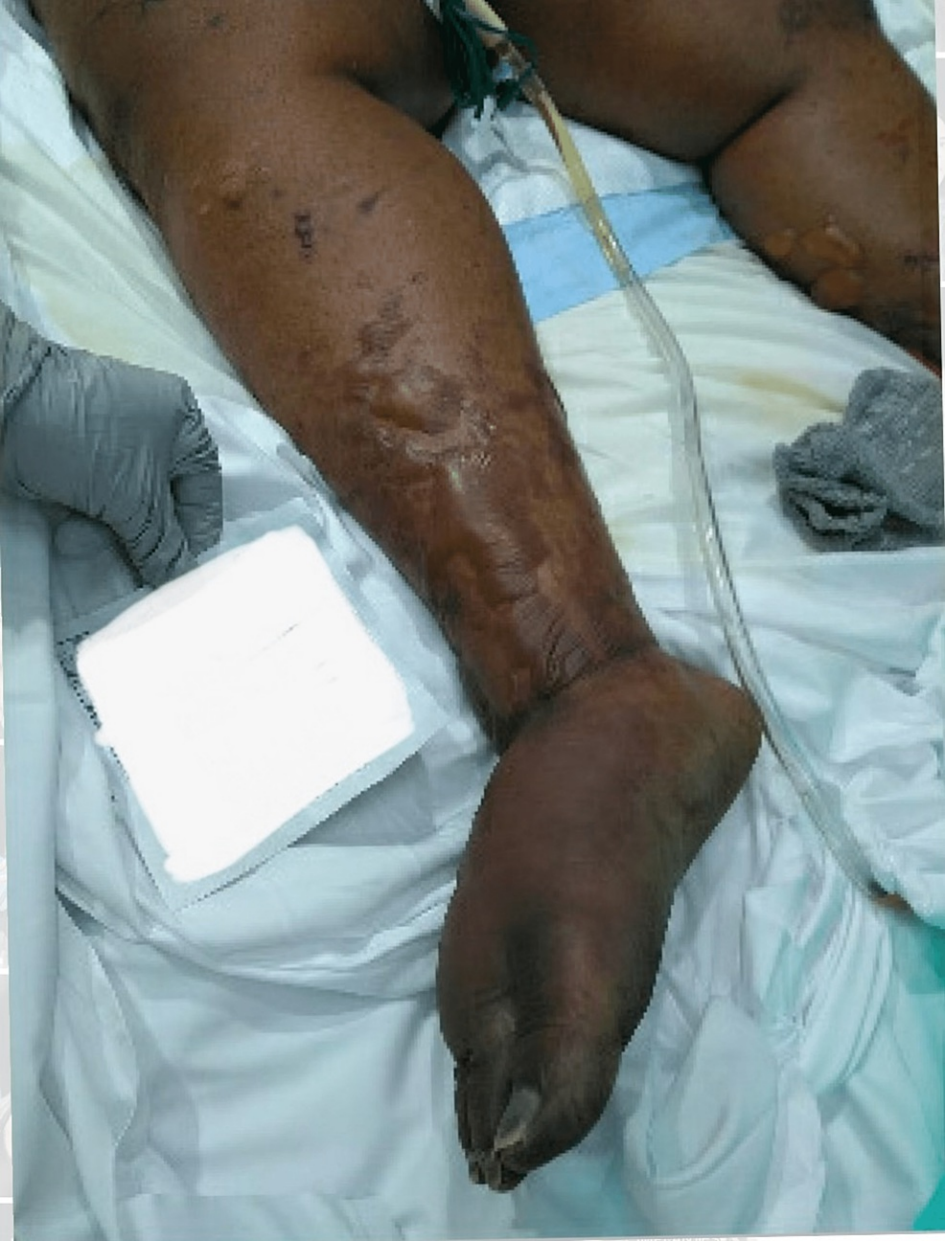
Right lower extremity with scattered bullae

A 50-year-old female with a past medical history of hypertension, asthma, obesity, and chronic cocaine abuse presented to the hospital by ambulance after being found unconscious in her home. The patient had been found by her brother who believed she had been lying in her own feces and urine for several days. The patient had a long history of daily cocaine use and was reported to have been seen inhaling cocaine on the morning of her admission. She did not take any prescribed over-the-counter medications, but she did smoke marijuana recreationally. Upon arrival at the emergency department, the patient was unalert and uncooperative; she was confused but withdrew her extremities to painful stimuli. The patient’s initial vitals were pertinent for a BMI of 54.59 and she was afebrile and hemodynamically stable. Physical examination showed absent bilateral dorsalis pedis, posterior tibialis, and radial pulses. Skin examination revealed diffuse purpura, scattered tense bulla (Figure 1), as well as an erosive lesion on the left thigh.

The patient had sloughing of dermal tissue with black discoloration of the feet and hands which were also cold to the touch. Upon inspection of her face, she had dried blood surrounding her mouth and nose, as well as brown mucoid discharge from both eyes with scleral icterus also present. Shortly after arrival, the patient’s mental status deteriorated, and she was intubated and admitted to the intensive care unit.

Complete blood count was remarkable for a white blood cell count of 15.3 × 10^9/L (Nl 4.5 to 11.0 × 10^9/L), and a platelet count of 25,000/mcl (Nl 150-450 x10^3/mcl) with schistocytes seen on blood smear. Blood cultures tested positive for *Staphylococcus epidermidis*. A transthoracic echocardiogram was performed which was negative for potential seeding of infection on cardiac valves. The degree of schistocytes warranted evaluation for thrombotic thrombocytopenic purpura with an ADAMTS13 level. However, clinical suspicion for this disorder remained low, hence pursuing plasmapheresis was not recommended.

Complete metabolic panel showed elevated levels of blood urea nitrogen at 105 mg/dL (Nl 7-18 mg/dL), potassium 5.7 mEq/L (Nl 3.5-5.2 mEq/L), creatinine 3.10 mg/dL (Nl 0.7-1.3 mg/dL), aspartate aminotransferase (AST) 330 U/L (10-36 U/L), alanine aminotransferase (ALT) 96 U/L (Nl 4-36 U/L), as well as a total bilirubin level of 4.9 mg/dL (Nl 0.1-1.2 mg/dL). The patient’s lactic acid level was 3.7 mmol/L with a creatine phosphokinase (CPK) of 11,000 mcg/L (Table 1). These laboratory values indicated that the patient was experiencing acute kidney injury with rhabdomyolysis as well as metabolic encephalopathy. Urine toxicology was positive for opioids and cocaine.

**Table 1:** Laboratory workup results

Laboratory Value	Result	Normal Range
White blood cell count	15.3 × 10^9^/L	4.5 to 11.0 × 10^9^/L
Platelet count	25,000/mcl	150,000 to 400,000/mcl
Blood urea nitrogen	105 mg/dL	7-18 mg/dL
Creatinine	3.10 mg/dL	0.7-1.3 mg/dL
Potassium	5.7 mEq/L	3.5-5.2 mEq/L
Alanine transaminase (ALT)	96 U/L	4-36 U/L
Aspartate transaminase (AST)	330 U/L	10-36 U/L
Bilirubin	4.9 mg/L	0.1-1.2 mg/dL
Lactic acid	3.7 mmol/L	<2 mmol/L
Creatine phosphokinase	11,000 mcg/L	10 to 120 mcg/L

Serology was positive for anti-ribonucleoprotein (anti-RNP) antibodies which prompted an antinuclear antibody (ANA) level to be ordered. The ANA level had a low titer of 1:40 and this, in conjunction with a negative lupus anticoagulant workup, allowed the specialists to have a low suspicion of a rheumatologic or autoimmune disease. Nonetheless, C3, C4, and immunoglobulin levels were ordered which were negative for pathology showing that the patient presentation was not consistent with a vasculitis or immunobullous disorder.

**Figure 2:**
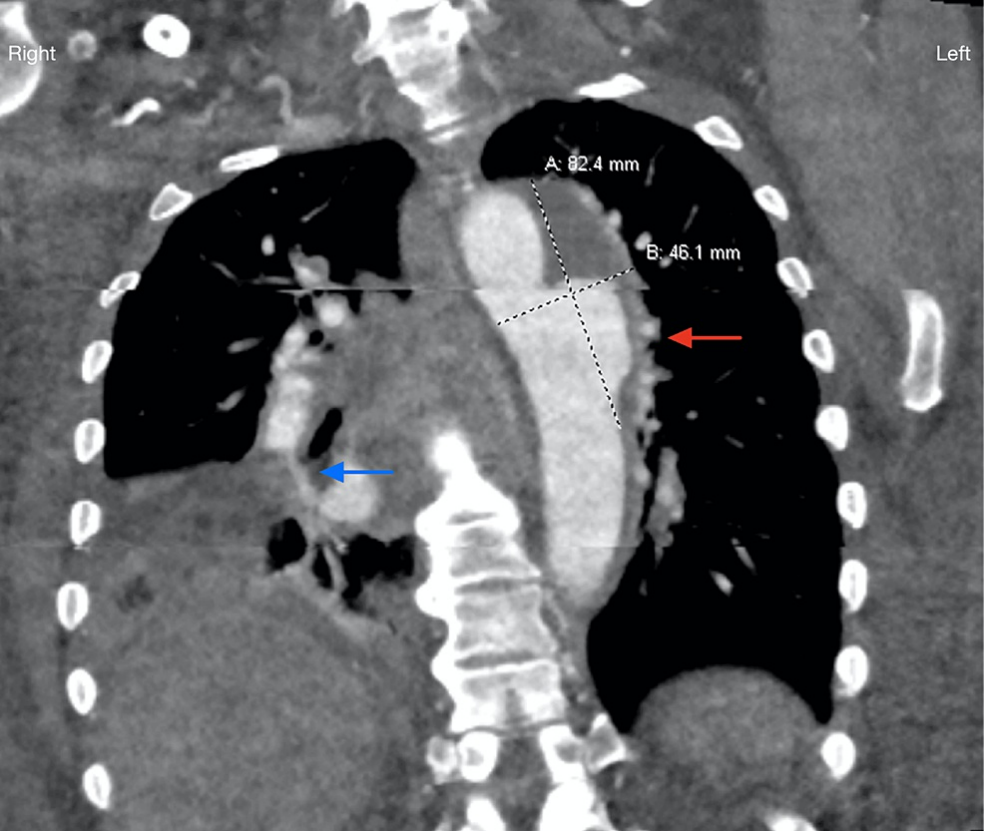
Computed tomography angiography of the chest showed an eccentric aneurysm involving the distal aortic arch and descending thoracic aorta partially thrombosed (red arrow). Multiple pulmonary emboli. Opacification in the right lung may reflect early infarction (blue arrow).

**Figure 3:**
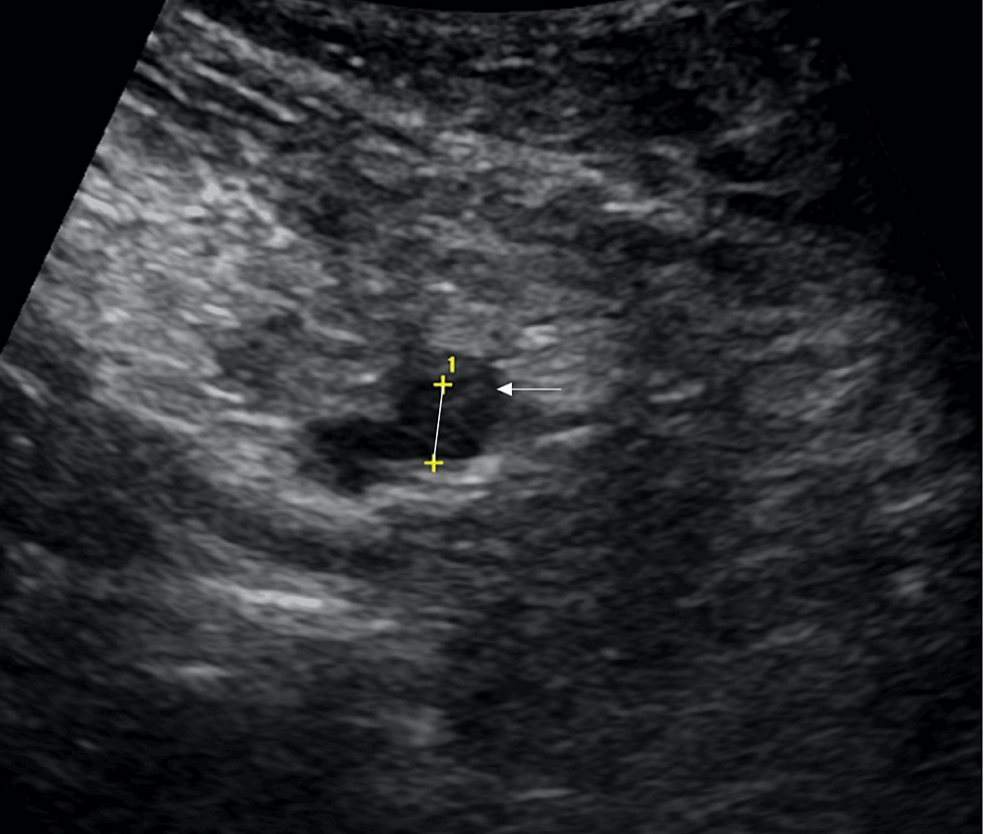
Doppler venous ultrasound with left popliteal deep venous thrombosis (white arrow).

Computed tomography (CT) of the lower extremities without contrast ruled out evidence of myonecrosis but showed nonspecific superficial soft tissue edema of the dorsal aspects of the bilateral feet describing blistering which correlated with visualization on physical exam. CT angiography of the chest revealed multiple pulmonary emboli, filling defects in the right atrium and right atrial appendage, and distal aortic arch and descending thoracic aorta partial thrombosis (Figure 2). Duplex ultrasonography revealed thrombosis in the right internal jugular vein and bilateral popliteal veins (Figure 3). Magnetic resonance imaging of the brain demonstrated supratentorial and infratentorial microhemorrhages. Skin biopsy of the left anterior thigh revealed epidermal separation and vascular thrombi, consistent with a bullous vasculopathy with retiform purpura.

In order to rule out intrinsic hematologic causes of coagulopathy, the following studies were ordered: Factor V Leiden, protein C&S deficiency, anti-thrombin deficiency, and anti-phospholipid antibody syndrome. These tests were negative and further testing for homocysteinemia was sent which showed a homocysteine level of 28.6 mcmol/L (Nl < 15 mcmol/L).

## Discussion

Cocaine produces vasoconstriction due to stimulation of alpha-adrenergic receptors, increasing the production of endothelin and decreasing the production of nitric oxide [6]. Cocaine-induced myocardial ischemia is characterized by an increase in myocardial oxygen demand, vasoconstriction of the coronary arteries, and an increase in platelet activation and aggregation with thrombi formation [6]. Although the mechanism of coronary artery involvement has been studied in detail, limited information has been presented on how cocaine use affects the peripheral vasculature.

Several hypotheses of cocaine being a pro-thrombotic agent have been proposed in addition to its vasospastic properties [7]. Heesch et al. in 2000 reported an increase in platelet factor 4 and β-thromboglobulin clotting factors in subjects after administration of pharmacological cocaine. It was found that even at small doses, cocaine caused an increase in platelet activation, platelet a-granule release, and microaggregate formation [8]. In another study by Siegal et al. in 1999, there was a significant increase in von-Willebrand factor after intravenous administration of cocaine at a dose of 0.4 mg/kg. The authors concluded that the increase in von Willebrand factor may precipitate an increase in platelet adhesion, aggregation, and intravascular thrombosis [9]. This same study also found a significant increase in the hemoglobin level, hematocrit, and red blood cell count and explained that this altered blood viscosity could also be contributing to the thrombogenic properties following cocaine use [9].

Many of the studies referenced in this article have limitations such as using in-vivo pharmacological cocaine versus illicit "street cocaine" [7] used by clinical studies involving patients such as the one in this case report. The literature also reports isolated cases of patients who present with major cardiovascular complications rather than complications involving vascular events regarding hypercoagulability-induced pathologies [10].

There are few studies that describe cocaine use in association with limb ischemia [4,11-15]. Moreover, ischemia involving all four extremities is not often reported [13,16,17]. Two possible described mechanisms of limb ischemia include the effect of bacterial infection and complement-induced thrombotic microangiopathy. Hoeger et al. described cocaine-induced tissue ischemia associated with *Streptococcus pyogenes group A* and *Staphylococcus aureus*. The authors described a possible synergy between the effects of bacterial proteases and cocaine-induced impairment of soft tissue perfusion [14]. Our patient’s blood culture was positive for *S. epidermidis* bacteremia and this, in combination with her chronic cocaine use, may explain the extensive ischemia that she developed. Another theory was proposed by Dejman et al. which suggested that complement activation in a patient with reduced level of serum complement C3 and normal C4 may contribute to the development of cocaine-induced thrombotic microangiopathy [18].

## Conclusions

Cocaine-associated thrombotic microangiopathy is infrequently reported but may provide an explanation for the ischemia involving all four extremities in our patient. The presentation was challenging due to the widespread complications of four-limb ischemia, pulmonary emboli, distal aortic arch and descending thoracic aorta partial thrombosis, deep venous thrombosis, acute kidney injury, and rhabdomyolysis. To our knowledge, this is the fourth case reported of four-limb ischemia implicated in chronic cocaine abuse. There are limited published cases in the literature which may be due to under-reporting, but this case report serves to add to the current literature.
